# Ideal Weight and Weight Discrepancy: A Study of Life Course Trajectories and Intercohort Change in the Netherlands

**DOI:** 10.3389/ijph.2024.1606278

**Published:** 2024-02-07

**Authors:** Liliya Leopold

**Affiliations:** ^1^ Department of Sociology, University of Amsterdam, Amsterdam, Netherlands; ^2^ Department of Sociology, University of Bamberg, Bamberg, Germany

**Keywords:** ideal weight, weight discrepancy, life-course trajectories, intercohort change, LISS

## Abstract

**Objective:** This study examined how individuals’ ideal weight and weight discrepancy (between ideal and actual weight) changed over the life course and across cohorts.

**Methods:** The study used population-representative longitudinal data collected in the Netherlands (*N* = 61,431 observations between 2007 and 2018 among *N* = 13,409 individuals aged 16 to 80 and born 1927–2000).

**Results:** Ideal weight increased linearly with age. Weight discrepancy showed a bell-shaped age pattern. Approximately half of the age-related increase in ideal weight was associated with concurrent increases in actual weight. Ideal weight and weight discrepancy increased slightly across cohorts. The cohort-related increase in ideal weight vanished after adjusting for change in actual weight. Analyses of population heterogeneity showed similar patterns of change in both outcomes across groups, although levels differed by gender, education, and migration status even after adjusting for differences in actual weight between these groups.

**Conclusion:** These results show that ideal weight and weight discrepancy in the Netherlands change substantially with age and modestly across cohorts. Potential explanations include changes in physical appearance and in the importance of physical appearance.

## Introduction

Weight discrepancy—the difference between actual and ideal body weight—is one of the central aspects of body image [1–6]. In approximately 70% of women and 50% of men in mainly Western study populations, individuals’ actual weight deviates from their ideal weight [3, 7]. Among women, thin bodies are idealized, and weight discrepancy is associated with a wish to lose weight [8, 9]. Among men, lean and muscular bodies are idealized, and weight discrepancy is often tied to muscularity, with men typically desiring to increase lean muscular body mass at the expense of fat mass [10]. A pronounced weight discrepancy can be a risk factor for disordered eating, depression, low self-esteem, and reduced life satisfaction in both sexes [11–13].

While previous studies have offered valuable insights, our knowledge about the nature of weight discrepancy is still limited especially in terms of individual and social change. Little is known about [1] how ideal weight and weight discrepancy *change with age*, as individuals move through their life course, adopt different social roles, and experience changes in their physical appearance; and [2] how ideal weight and weight discrepancy *change across cohorts*, as people experience different beauty ideals, levels of common body weight among peers, and shifting social body weight norms?

Previous research suggests that weight perceptions develop through social comparisons within reference groups defined by gender, age, and ethnic background, whereby individuals’ ideas about which weight is ideal and the extent to which their actual weight matches this ideal are influenced by what is prevalent and what is considered ideal, normal, and socially accepted in these groups [14–16]. Furthermore, individuals’ beliefs about ideal body weight have been consistently linked to their actual weight and to the pressures that individuals experience when exposed to cultural ideals presented in the media [3, 17, 18]. Each of these factors is subject to change. Over an individual life course, average adult body weight increases substantially [19]. Marked increases are also found across cohorts, as documented for various societies across recent decades [19, 20]. Furthermore, over the course of the 20th century, cultural ideals for body weight have shifted from fuller to increasingly slimmer or even underweight bodies [17]. However, recent research suggests that this trend may have reversed, as evidenced by the emergence of body positivity and body neutrality movements and greater body size diversity presented in the media [2]. Finally, studies on trends in social body weight norms also suggest that higher levels of body weight became more socially accepted [21, 22]. Although this evidence demonstrates that most determinants of weight perceptions undergo substantial change, little is known how about how these perceptions, and the resulting perceived discrepancy between ideal and actual weight, have changed.

Most previous studies have looked at age patterns in related outcomes of weight perception and (dis)satisfaction with weight and reported patterns of stability in women and mixed patterns in men (refer to reviews [23, 24]). However, most of these studies were based on cross-sectional data and assumed no variation across cohorts. This assumption is challenged by evidence on fluctuations in cultural ideals, the rise in body weight among younger cohorts, and changes in social body weight norms [8, 14, 18, 19, 21, 22].

To date, only one study from New Zeeland has delved into how body image satisfaction, a related but broader construct encompassing satisfaction with weight, size, and shape, changed with age while also considering intercohort variations [25]. Contrary to most previous cross-sectional studies, this study revealed that body image satisfaction increased with age for both sexes. Moreover, supporting the expectation of change across cohorts, this study found that the growth in satisfaction was steeper in newer cohorts, especially among women. Other studies using data from U.S. and the U.K. showed that the Body Mass Index (BMI) of individuals who self-classified their weight as being in the normal range has risen considerably in younger cohorts and more recent periods [20, 21]. These findings underscore the need to consider potential changes across cohorts when studying age patterns of ideal weight and weight discrepancy.

Consequently, present study aimed at studying change in perceptions of ideal weight and the associated weight discrepancy over individual life courses and across cohorts. Population-representative data analyzed for this study came from twelve waves (2007–2018) of the Longitudinal Internet Studies for the Social Sciences (LISS) panel in the Netherlands. Using these data, I assessed, first, mean changes in ideal weight and weight discrepancy with age and across cohorts, and second, the role of changes in individuals’ actual weight and of body weight in reference groups to gain initial insight into potential mechanisms underlying age and cohort change.

## Data, Measures and Methods

The present study was pre-registered at the Open Science Framework (OSF). The pre-registration document and the replication files are available at the author’s OSF profile [26] and personal web page.

### Data

Studying how ideal weight and weight discrepancy develop over the life course and change across cohorts requires population-representative longitudinal data covering a wide age range on multiple birth cohorts with multiple measures of ideal weight and current weight. The LISS panel is one of the few surveys worldwide that fulfills these requirements. LISS is a probability-based online survey representative for the non-institutionalized population of the Netherlands aged 16 and older. Respondents that do not have Internet access are loaned a computer, a method developed especially for elderly people without computer experience [27]. The LISS panel does not include sample weights. To ensure representativeness, LISS collected refreshment samples in 2009, 2012, 2014, and 2017. Information on data availability is included in Appendix 1. Data on ideal weight and current weight were collected in 12 waves from 2007 to 2018. This provided exceptionally high quality of data for analyses of life course trajectories compared to previous research covering only a 5-year period [25].

### Sample Selection

I restricted the sample to respondents who participated in at least one of the 12 waves in which questions about ideal weight and current weight were included. This resulted in an initial sample size of 13,693 respondents with 63,603 observations (i.e., person-years). Despite the overall high quality of LISS data, age-related response bias was present in the recruitment phases [28]. Despite refreshment efforts, sampling elderly adults remains challenge in LISS—similar to other probability-based online panels. To limit response and survivor bias, I restricted the age range to 16—80 years, corresponding to cohorts born between 1927 and 2002 (N = 13,529 individuals with N = 62,120 observations). Further, and in line with previous research [19], I removed observations with implausible values of BMI and ideal BMI (<10 kg/m^2^ or >90 kg/m^2^). This resulted in a final analytic sample size of N = 13,409 respondents with N = 61,431 observations.

### Measures

#### Ideal Weight

The measure of ideally desired weight was based on survey question “What is your target weight?” This formulation is a variation of common formulations to determine which weight individuals ideally desire. In line with previous research, I combined information on ideal weight with information on self-reported height to create an ideal BMI variable using the standard formula (3). Information used to calculate ideal BMI was collected yearly from 2007 until 2018. Average ideal BMI was 23.4 (SD = 3.3) for women and 24.5 (SD = 2.7) for men (see Table 1).

**TABLE 1:** Descriptive statistics. Netherlands 2007–2018.

	Women					Men				
	*M*	*%*	*SD*	*Min*	*Max*	*M*	*%*	*SD*	*Min*	*Max*
Age & Cohort										
Age	47.66		16.74	16	80	49.94		16.86	16	80
Year of birth	1967		17.78	1927	2002	1965		18.05	1927	2002
Education (highest level attended)										
<= Intermediate secondary		28		0	1		23		0	1
Higher second. & interm. vocational		31		0	1		31		0	1
Higher vocational		27		0	1		27		0	1
University		14		0	1		18		0	1
Civil status										
Married		54		0	1		59		0	1
Divorced/Separated		10		0	1		9		0	1
Widowed		6		0	1		3		0	1
Never married		29		0	1		29		0	1
Immigration status										
No migration background		84		0	1		84		0	1
1st generation migrant		3		0	1		4		0	1
2nd generation migrant		13		0	1		12		0	1
Body weight & height										
BMI (kg/m^2^)	25.28		4.98	10.98	81.37	25.71		3.99	13.88	64.40
Height (cm)	168.21		6.71	140	196	180.91		7.61	148	210
Weight (kg)	71.53		14.53	40	189	84.15		14.16	43	190
Ideal weight and weight discrepancy										
Ideal BMI (kg/m^2^)	23.40		3.33	10.41	73.58	24.46		2.72	13.23	88.16
Weight discrepancy (kg/m^2^)	2.06		2.81	0	51.07	1.59		2.16	0	65.30
Weight discrepancy (%)	7.19		8.01	0	211.11	5.66		6.76	0	326.91
Current BMI = Ideal BMI		18		0	1		21		0	1
Current BMI > Ideal BMI		76		0	1		67		0	1
Current BMI < Ideal BMI		6		0	1		12		0	1
Attrition										
Left panel before 2018		59					59			
Number of waves	5.23		4.01	1	12	5.31		4.14	1	12
*N* individuals (Observations)	7,269 (33,085)					6,140 (28,346)				

Note. Data from 12 waves of the Longitudinal Internet Studies for the Social Sciences (LISS) collected between 2007 and 2018. *M* refers to the mean, *%* refers to the share of the observations coded as 1 in 1–0 coded variables, *SD* refers to the standard deviation, *Min* refers to the minimum value observed in the data. *Max* refers to the maximum value observed in the data. Descriptive statistics on time-varying variables are averaged over observations. Descriptive statistics on time-constant variables are averaged over individuals.

#### Weight Discrepancy

Weight discrepancy was measured as the degree of discordance between ideal BMI and current BMI, calculated from self-reports of weight and height using the standard BMI formula (i.e., weight in kilograms divided by squared height in meters) [3, 29].

I constructed a continuous indicator of *absolute* weight discrepancy, which took the value of 0 if ideal weight equaled current weight. The variable was coded positive for all deviations from current weight, regardless of direction (i.e., whether individuals wished to weigh less or more). Deviations in women were almost exclusively due to the wish to weigh less. Men showed a more heterogeneous pattern, as deviations at younger age were mainly due to the wish to weigh more. The measure of weight discrepancy was available annually from 2007 until 2018. On average, women’s ideal BMI deviated by approximately 2 kg/m^2^ from their actual BMI; men’s ideal BMI deviated by approximately 1.6 kg/m^2^ from their actual BMI (Table 1). Removing outliers (those with the deviation of ideal weight from actual weight larger that 15 kg/m^2^, which applied to about 0.4% of the sample) did not change any of substantive findings. Thus, the outliers remained in the analytic sample.

Next to the measure of absolute weight discrepancy and following previous studies, I constructed an indicator of weight discrepancy in percent, indicating the relative deviation of actual body weight from ideal body weight. This variable took the value 0 if ideal BMI equaled actual BMI. Values higher than zero indicated a relative deviation of actual body weight from ideal body weight in percent, regardless of the direction of the deviation. For instance, a deviation of 2 kg/m^2^ from an actual BMI of 20 kg/m^2^ was measured as a 10% deviation. The substantive interpretation of this measure was that individuals would have to change 10% of their BMI to reach their ideal.

In addition to these two continuous measures, I analyzed categorical measures to gain further insight into the direction of weight discrepancy. First, actual and ideal weight being fully *concordant* (actual = ideal) was coded 1 and 0 otherwise. This applied to approximately 20% of observations both among men and women. Second, the *wish to weigh more* (actual < ideal) was coded as 1 if ideal weight was higher than current weight and 0 otherwise. This applied to 6% of observations of women and 12% of observations of men. Finally, the *wish to weigh less* (actual > ideal) was coded 1 if ideal weight was lower than current weight and 0 otherwise. This applied to 76% percent of observations of women and 67% of observations of men (Table 1).

#### BMI

The measure of Body Mass Index (BMI) was based on annual self-reports of weight and height and calculated using the standard BMI formula. Measures of BMI based on self-reported weight and height are sufficiently accurate and correspond closely with BMI based on measured weight and height, although weight is usually slightly under-reported, and height is usually over-reported [30]. The inaccuracy increases with BMI and is more pronounced among older adults, mainly due to over-reporting height. Although self-reported BMI is less accurate than measured BMI, it had an important advantage for the purposes of the present study: As BMI was mainly used to identify actual-ideal weight discrepancy—an individual’s *perception* of the degree to which actual weight deviated from ideal weight—subjectively perceived weight was a more pertinent measure than objectively measured weight.

Next to using individuals’ BMI to measure actual-ideal weight discrepancy, BMI was also used (i) as a time-varying variable to assess the effect of *changes* in individual BMI, and (ii) as a time-constant variable to assess effect of differences in BMI across reference groups. Following the approach suggested by Burke et al. (2010), an indicator of *reference group BMI* was constructed as average BMI within birth year, sex, and migration status.

#### Sex

All analyses were performed separately for men and women. Information on sex was obtained via self-reports.

#### Age and Cohort

Age was defined as survey year minus birth year (range 16–80 years). Birth cohort ranged between 1927 and 2002. For the statistical models, different functional forms of age and cohort were tested to obtain the best model fit for different outcomes for men and women. The exact specifications are described below. Model fit analyses are included in Supplementary Appendix SA1 (Supplementary Material SA1). In all models presented in Tables 2, 3, age, cohort, and their polynomials were centered at their sex-specific means (see alsoSupplementary Tables SA4–SA6 in Supplementary Appendix SA3).

**TABLE 2:** HLM Models for Change in Ideal BMI for men and women. Netherlands 2007–2018.

	M1a	M1b	M1c	M1d
	Ideal BMI (kg/m^2^) Women	Ideal BMI (kg/m^2^) Men	Ideal BMI (kg/m^2^) Women	Ideal BMI (kg/m^2^) Men
Age	0.735***	0.447***	0.402***	0.218***
	[0.657, 0.812]	[0.374, 0.519]	[0.343, 0.461]	[0.165, 0.271]
Age2	−0.849***	−1.180***	−0.017	−0.449**
	[−1.263, −0.436]	[−1.562, −0.798]	[−0.338, 0.305]	[−0.740, −0.157]
Cohort	0.261***	0.254***	0.042	0.091***
	[0.187, 0.335]	[0.188, 0.320]	[−0.012, 0.097]	[0.041, 0.140]
Cohort * Age	−0.061*	−0.018	−0.031	−0.050**
	[−0.116, −0.005]	[−0.068, 0.031]	[−0.068, 0.005]	[−0.083, −0.017]
Cohort * Age2	−0.334***	−0.446***	−0.091**	−0.148***
	[−0.433, −0.234]	[−0.536, −0.357]	[−0.154, −0.028]	[−0.205, −0.092]
Indiv. BMI			0.457***	0.469***
			[0.451, 0.463]	[0.462, 0.475]
Intercept	23.648***	24.818***	23.408***	24.476***
	[23.557, 23.739]	[24.739, 24.898]	[23.361, 23.454]	[24.432, 24.519]
*N*	33,085	28,346	33,085	28,346

*Note*. Data from 12 waves of the Longitudinal Internet Studies for the Social Sciences (LISS) collected between 2007 and 2018. HLM refers to Hierarchical Linear Regression Models. Age, Age-squared, and Cohort were centered at sex-specific means and divided by 10. BMI was centered at sex-specific means. 95% confidence intervals in brackets. *p < 0.05, **p < 0.01, ***p < 0.001.

**TABLE 3:** HLM Models for Change in Weight Discrepancy in kg/m^2^ and in % for Men and Women. Netherlands 2007–2018.

	M2a	M2b	M3a	M3b
	Weight discr.(kg/m^2^) Women	Weight discr.(kg/m^2^) Men	Weight discr.(%) Women	Weight discr.(%) Men
Age	−0.014	0.114***	−0.248*	0.185
	[−0.090, 0.063]	[0.055, 0.173]	[−0.479, −0.017]	[−0.007, 0.378]
Age2	−0.788***	−0.558***	−2.577***	−1.481***
	[−1.219, −0.356]	[−0.745, −0.372]	[−3.881, −1.273]	[−2.101, −0.861]
Cohort	0.174***	0.156***	0.385***	0.511***
	[0.099, 0.249]	[0.092, 0.220]	[0.164, 0.607]	[0.307, 0.715]
Cohort2		−0.107		
		[−0.314, 0.101]		
Cohort * Age	−0.013		−0.091	
	[−0.063, 0.038]		[−0.240, 0.058]	
Cohort * Age2	−0.151**		−0.265	
	[−0.241, −0.060]		[−0.532, 0.002]	
Intercept	2.289***	1.823***	7.738***	6.217***
	[2.211, 2.367]	[1.758, 1.888]	[7.524, 7.953]	[6.022, 6.412]
*N*	33,085	28,346	33,085	28,346

Note. Data from 12 waves of the Longitudinal Internet Studies for the Social Sciences (LISS) collected between 2007 and 2018. HLM refers to Hierarchical Linear Regression Models. Age, Age-squared, and Cohort were centered at sex-specific means and divided by 10. BMI was centered at sex-specific means. 95% confidence intervals in brackets. *p < 0.05, **p < 0.01, ***p < 0.001.

#### Control Variables

Previous research has shown that weight perceptions may differ between demographic groups even after accounting for differences between groups in their actual weight, height, and BMI [21, 31]. In additional analyses, I examined potential socio-demographic differences by including controls for education, migration status, and civil status. The description of control variables is included in Supplementary Appendix SA1 (Supplementary Material SA2). Results on these additional analyses are presented below and in Supplementary Appendix SA3 and Supplementary Appendix SA4.

#### Missing Data

Item nonresponse was negligible, affecting less than 1% of observations on all variables. Panel attrition was substantial, as approximately 60% of respondents did not participate in the last wave in 2018. Panel attrition in LISS is similar to attrition in other panel surveys. A comparison of dropouts to non-dropouts showed similar BMI, ideal BMI, and actual-ideal weight discrepancy (Supplementary Tables SA2, SA3 in Supplementary Appendix SA2). Dropouts were younger than non-dropouts due to shorter observation times. Overall, the analysis of missing data indicated that systematic nonresponse or attrition were no sources of bias in the present study.

### Statistical Analysis

#### Analytic Strategy

The analysis was based on cohort-sequential growth curve models, utilizing within-person and between-person variation to estimate trajectories of ideal weight and satisfaction with weight over the life course and intercohort change in these trajectories [32–34]. In this design, age growth curves estimates are based on within-person variation during the observation window of up to 12 years. Intercohort change estimates are based on variation in initial levels and in between-person variation in within-person age growth curves. The length of individual observation periods and the range of birth cohorts are the key quality requirements for data supporting this type of analysis. Supplementary Table SA1 in Supplementary Appendix SA2 shows age overlaps between cohorts in the analytic sample, demonstrating the high quality of LISS data for these purposes.

Based on this data structure, hierarchical linear regression models (HLM) were used to simultaneously estimate within-person change with age, between-person change across cohorts, and their interactions [35–37]. Based on these models, the main analyses assessed changes with age and across cohorts in ideal weight and weight discrepancy for women and men. In a series of additional analyses, I included indicators of change in individual BMI (M1c and M1d in Table 2; M1e-M1h in Supplementary Table SA4 in Supplementary Appendix SA3), BMI in reference groups (M1e-M1h, Supplementary Table SA4 in Supplementary Appendix SA3), and demographic controls (M1g-M1h, Supplementary Table SA4; M2c and M2d, M3c and M3d in Supplementary Table SA5 in Supplementary Appendix SA3). A detailed description of the models and model fit analyses is included in Supplementary Material SA1 in Supplementary Appendix SA1.

## Results

**FIGURE 1:**
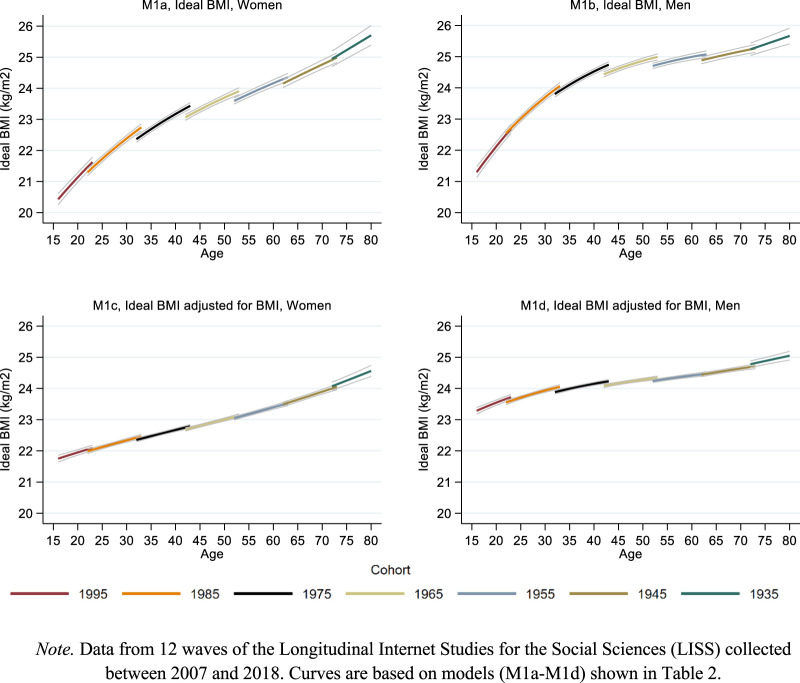
Life course and cohort profiles of ideal weight. Note. Data from 12 waves of the Longitudinal Internet Studies for the Social Sciences (LISS) collected between 2007 and 2018. Curves are based on models (M1a–M1d) shown in Table 2. Netherlands 2007–2018.

**FIGURE 2:**
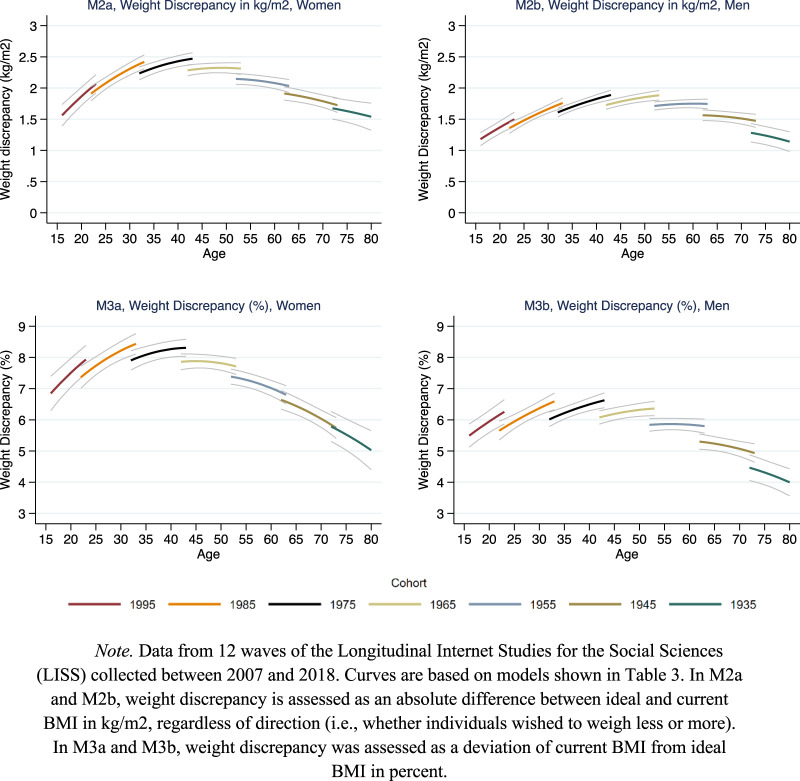
Life course and cohort profiles of weight discrepancy. Note. Data from 12 waves of the Longitudinal Internet Studies for the Social Sciences (LISS) collected between 2007 and 2018. Curves are based on models shown in Table 3. In M2a and M2b, weight discrepancy is assessed as an absolute difference between ideal and current BMI in kg/m^2^, regardless of direction (i.e., whether individuals wished to weigh less or more). In M3a and M3b, weight discrepancy was assessed as a deviation of current BMI from ideal BMI in percent. Netherlands 2007–2018.

**FIGURE 3:**
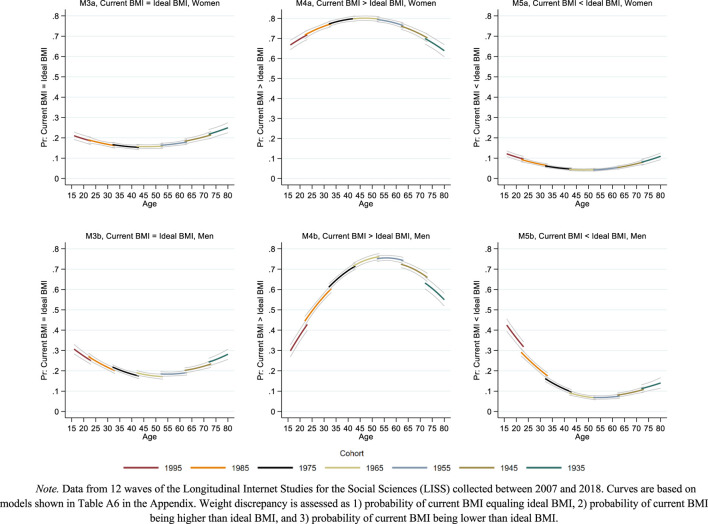
Life course and cohort profiles of weight discrepancy. Note. Data from 12 waves of the Longitudinal Internet Studies for the Social Sciences (LISS) collected between 2007 and 2018. Curves are based on models shown in Supplementary Table SA6 in the Supplementary Appendix. Weight discrepancy is assessed as 1) probability of current BMI equaling ideal BMI, 2) probability of current BMI being higher than ideal BMI, and 3) probability of current BMI being lower than ideal BMI. Netherlands 2007–2018.

Figures 1–3 present the main results in graphical form. The models behind the predictions presented in these figures are shown in Tables 2, 3, and Supplementary Table SA6 (Supplementary Appendix SA3). To simplify the presentation of the results in Figures 1–3, the cohort variable was fixed at specific values (1995, 1985, 1975 etc.) showing age curves for every tenth cohort from 1935 to 1995. The length of each curve was defined by the age range in which a birth cohort was observed. The gaps between the curves at overlapping ages indicate cohort effects: In the absence of cohort effects, age curves connect; in presence of cohort effects, age curves disconnect and the overall pattern appears ragged [38, 39]. Gray thin lines surrounding each curve indicate 95% confidence intervals.

As visible from the upper plots in Figure 1, ideal BMI increased with age for men and women. For both sexes, this increase was approximately linear until age 40 and flattened thereafter, especially in men. The life course increase of ideal BMI was substantial. In women, ideal BMI increased from 21 kg/m^2^ at age 20 to approximately 25 kg/m^2^ at age 70, and even further at older ages. In men, ideal BMI increased from 22 kg/m^2^ at age 20 to approximately 25 kg/m^2^ at age 70. Intercohort change in ideal BMI was modest and only found in women, with a tendency towards higher ideal BMI across cohorts.

The bottom plots of Figure 1 show how these estimates changed after adjusting for changes in actual BMI. The resulting curves can be interpreted as the estimated age and intercohort change in ideal BMI if actual BMI remained constant. In these plots, BMI was fixed at sex-specific means. Both plots show that although ideal BMI still increased with age, this increase was slower and smaller in scope as compared to the unadjusted models shown in the upper plots. In both women and men, approximately half of the age-related increases in ideal BMI were associated with concurrent increases in actual BMI. The slight cohort trends in the unadjusted models vanished after adjusting for actual BMI.

In additional analyses, presented in Supplementary Table SA4 (Supplementary Appendix SA3), I controlled for a time-constant indicator of average BMI in reference groups (models M1e and M1f) and included demographic controls (models M1g and M1h). Although there was a small positive association between higher reference group BMI and ideal BMI and a negative association between education and ideal BMI, patterns of change with age and across cohorts remained similar (see Supplementary Figure SA1 in Supplementary Appendix SA4).

Figure 2 shows the results on weight discrepancy, assessed as deviations of actual weight from ideal weight coded as positive absolute BMI-point values (Figure 2, upper panels) and as relative deviations of actual BMI from ideal BMI in percent (Figure 2, lower panels). The curves show a bell-shape for absolute weight discrepancy over the life course both among men and women. Weight discrepancy peaked in midlife (ages 40–50) while it was lower in young adulthood and in older age. At the midlife peak, average deviations of actual weight from ideal BMI amounted to approximately 2.5 points in women and slightly less than 2 points in men. As visible from the disconnected age lines, results for both women’s and men’s weight discrepancy showed a slight but consistent growth across cohorts. The results on relative weight discrepancy showed similar trends, although the decline in older age was steeper and reached even lower levels than in young age.

Results from additional analyses controlling for demographic indicators (Supplementary Table SA5, Supplementary Appendix SA3) showed higher absolute and relative weight discrepancies for lower education levels and for people with migration background as compared to natives. There were no differences related to civil status. Adding demographic controls to the models lowered the levels of weight discrepancy while the patterns of change with age and across cohorts remained similar (Supplementary Figure SA2, Supplementary Appendix SA4).

Figure 3 shows change in categories of weight discrepancy for women (top plots) and men (bottom plots), adding detail about the direction of deviation between actual and ideal weight. For women, the probability of full concordance between actual and ideal weight (i.e., actual BMI = ideal BMI) remained almost constant around 20% across age and cohort. For men, this probability was similar to women’s in midlife and around 10 percentage-points higher in younger and older age.

Women’s wish to weigh less (i.e., actual BMI > ideal BMI) peaked at a probability of 80% in midlife (age 50). In younger and older age, women’s wish was somewhat less pronounced but still prevalent at estimated levels around 60%–70%. In men, the wish to weigh less was less prevalent and showed more variation over the age range studied. A sharp increase from approximately 30% in teenage men to 75% in men aged 50 was followed by a decline from age 60 onwards, approaching a level of 50% at age 80.

Women’s wish to weigh more (i.e., actual BMI > ideal BMI) remained rare throughout the age range, slightly exceeding 10% only at younger and older ages. Again, men showed more change with age, particularly in younger adulthood. Approximately 40% of teenage men wished to weigh more, a probability that dropped to and remained around 10% from age 45 onwards. The results for women and men indicated no sizable cohort effects in any of these three additional outcome measures of weight discrepancy.

## Discussion

This study examined change in ideal weight and in weight discrepancy among men and women. Longitudinal data from the LISS panel captured wide ranges of age and cohort, allowing to assess individual change over the life course and social change across cohorts in these two dimensions of body image.

The results showed that ideal weight increased with age both among men and women. Half of the age-related increase in ideal weight vanished after adjusting for change in actual weight, suggesting that individuals gradually adjusted their ideal weight to increases in their actual weight. Although the direction of this relationship remains somewhat contentious (individuals could also adjust their actual weight towards their increasing ideal weight), further results demonstrated that increases in ideal weight were accompanied by an increase in the wish to weigh less and with a decrease in the wish to weigh more observed across most of the adult life course.

Although half of the age-related increase in ideal weight was associated with increases in actual weight, it is important to note that the other half was not. Moreover, this unexplained part of the age increase in ideal weight remained even after adjusting for BMI in reference groups and for demographic controls. Possible explanation are life-course changes in beauty ideals and in the importance of physical appearance [24]. For example, having a fuller figure may be considered more beautiful or more appropriate in social roles adopted in middle and older age. In this regard, research has suggested that women’s ideal weight increases after motherhood. In older age, thinness may have shifted from a signal of beauty and health to a signal of frailty and sickness [7, 24].

These potential explanations for increasing ideal weight also suggest that weight discrepancy may decrease with age, as beauty standards shift and physical appearance becomes less important. The present study shows, however, that trajectories of ideal weight and trajectories of weight discrepancy converged until midlife, as increases in actual body weight exceeded increases in ideal weight. Weight discrepancy was most pronounced in mid-adulthood—the age at which ideal weight peaked. Weight discrepancy declined only thereafter, while the increase in ideal weight flattened out.

The mid-life stage—in which the present study has identified the peak of weight discrepancy—has received the least attention in previous research, which focused primarily on young people and, more recently, on older people [3, 7]. The finding of a mid-life peak in weight discrepancy points to a previously undetected vulnerable life stage in terms of body image: A high discrepancy between actual and ideal weight constitutes a source of stress and a risk factor for physical and mental health problems [29]. Future research is needed to shed further light on the causes, extent, and potential implications of weight discrepancy in midlife. Potential causes are accelerated weight gain, changes in body core temperature, and related challenges of adjustment in terms of physical activity and calory intake [40]. Among women, changes in body image related to menopause might constitute a further potential explanation for rises in weight discrepancy [41].

For social change across birth cohorts (1930–2000), results showed a slight increase in ideal weight and a moderate increase in weight discrepancy. The slight intercohort increase in ideal weight vanished when adjusting for changes in actual BMI. These results suggest that across cohorts, ideal BMI has increased as people became heavier on average, while heavier people tended to report higher ideal weight [3]. Similar to differences in life course patterns between the two dimensions of body image, the intercohort increase in ideal weight did not imply lower levels of weight discrepancy across cohorts. On the contrary, weight discrepancy increased in more recent cohorts. These results contradict ideas articulated in the literature expecting ideal weight to increase and weight discrepancy to decrease with trends towards higher body weight, weakening social body weight norms, and more body acceptance [2, 20, 21, 25]. One possibility is that these cultural shifts are less pronounced or less influential than hypothesized—generally or at least in the Dutch context of the present study. Another is that these cultural shifts—even if pronounced and influential—are offset or outweighed by countervailing factors such as increasing health knowledge. Across cohorts, individuals may have become more aware of the importance of a healthy lifestyle and the specific health risks related to higher body weight [42, 43]. With respect to intercohort increases in weight discrepancy, increasing awareness of health risks related to overweight and obesity might thus prevail over opposite effects related to increasing body acceptance.

Regarding differences between women and men, results showed largely similar patterns of life-course and intercohort change. However, women and men differed in the levels around which these changes unfolded. In women, ideal weight and satisfaction with weight was lower. In men, the wish to weigh less became dominant from age 30 onwards, whereas in women, the wish to weigh less was dominant over the entire life course. Consistent with previous research, men’s wish to weigh more was more prevalent than women’s. In addition to these established findings, the present study showed that gender differences in the wish to weigh more were limited to younger adulthood. Although no measures for the wish of becoming more muscular were available in the data, these results are consistent with studies on young men who often wish to increase lean mass [13]. From age 40 onwards, the gender gap narrowed and the wish to weigh less prevailed in both men and women. These results show that weight discrepancy and specifically the wish to weigh less is not a largely female phenomenon and needs to be addressed and monitored in men and in women.

When evaluating the results of the present study, it is important to consider its’ limitations. Specifically, the indicator of weight discrepancy was limited in measuring the level of subjective satisfaction with weight. Although weight discrepancy correlates with subjective satisfaction with weight, some studies have shown that a discrepancy between ideal and actual weight does not necessarily coincide with body weight dissatisfaction [5]. For the present study’s results, this suggests a possible alternative interpretation whereby the peak of weight discrepancy in midlife may primarily reflect a desire to improve health rather than dissatisfaction with the appearance of one’s body. A study from New Zealand employing a design similar to the present study but using subjective measures of satisfaction with weight, height, and shape found that satisfaction with weight did not decrease in midlife [25]. However, another longitudinal study from the USA [44] examining subjective satisfaction with weight found a decline between age 15 and 30, similar to what was observed in the present study. Based on the available data and evidence, it is not possible to assess whether these mixed results are due to differences in national contexts, in the nature of measurements, or in study designs.

Future international comparative studies based on long-term panels and covering a wide range of cohorts and countries are needed to shed further light on individual and social changes in ideal weight, weight discrepancy, and subjective satisfaction with body weight.
