# Supplementary material for: Ideal Weight and Weight Discrepancy: A Study of Life Course Trajectories and Intercohort Change in the Netherlands
Source: Int J Public Health. 2024 Feb 7;69:1606278. doi: 10.3389/ijph.2024.1606278 (PMC10880023; doi:10.3389/ijph.2024.1606278)
Supplement: Supplementary file 1 [file DataSheet1.pdf]

Appendix 1

Data availability

Each of the data sets used in the present study is available to the scientific community upon registration as a data user with LISS. Variables used in the present study are included in the sections “Background variables” (for birth year and gender), “Health” (BMI and ideal BMI) and “Religion and Ethnicity” (for identifying respondents with immigrant background).

Data Access

There are no restrictions to accessing the dataset for the scientific community.

Data Identifiers

All data sets used in the present study can be downloaded here (upon registration with LISS):

https://www.dataarchive.lissdata.nl/study_units/view/1

Access Date

I downloaded the data sets on 31.01.2022

Data Collection Procedures

The data collection procedure is described here: https://www.lissdata.nl/about-panel

Codebook

The codebooks for the background variables can be found here:

https://www.dataarchive.lissdata.nl/study_units/view/322

The codebooks for the variables used to construct BMI and ideal BMI can be found here:

https://www.dataarchive.lissdata.nl/study_units/view/13

Supplement S1: Statistical modelling

The HLM estimation allows within-person age trajectories to differ in their starting levels (random intercepts) and rates of change (random slopes). The estimation of HLM provides information about mean ideal weight and satisfaction with weight trajectories (growth curves) as well as individual variation around the average curves. The models allow data to be unbalanced in time and incorporate all respondents, regardless of the number of waves in which they were observed.

The growth curves for each of the outcomes *Y* (ideal weight and weight discrepancy) of respondent *i* at time *t* are calculated as follows (see 13, 26):

Level 1:

(1)

$$Y_{it} = \pi_{0i} + \pi_{1i} age_{it} + e_{it}$$

where $i = 1, \ldots, N$ are individuals in the sample, π_{0i} is an individual-specific intercept, and π_{1i} is the growth rate for individual *i*. This model estimates the intercepts (π_{0i}) and slopes (π_{1i}) of Level-1 variables.

Level 2:

(2)

$$\pi_{oi} = \beta_{00} + \beta_{01} Cohort + r_{0i},$$

$$\pi_{1i} = \beta_{10} + \beta_{11} Cohort_i + r_{1i}$$

where β_{pq} are the effect of cohort on intercept π_{oi} and slope π_{1i}, and r_{pi} is an error term for unmeasured time-constant characteristics of individual *i*.

Combining (1) and (2) yields:

(3)

$$Y_{it} = [\beta_{00} + \beta_{10}Age_{it} + \beta_{01}Cohort_i + \beta_{02}Cohort_i * Age_{it}] + [e_{it} + r_{0i} + r_{1i}age_{it}].$$

All analyses were performed separately for men and women. The parametrizations of age and cohort effects on each of the outcomes were based on three criteria, (a) similarity between observed and fitted data examined by diagnostic plots, (b) BIC comparison between models, and additionally (c) model parsimony if models were similar on criterion (a) and did not differ by more than 10 BIC points (28). This resulted in different functional forms of age and cohort and interactions between these two variables for different outcomes as well as slightly different functional forms for men and women. Inclusion of multiple polynomials of age and cohort terms as well as interaction terms between them complicates a straightforward interpretation of regression model coefficients. Therefore, studies on life course trajectories and intercohort change in physical and mental health outcomes (13, 29, 30) typically visualize their results as age-vector graphs, which allow for a detailed inspection of life course and intercohort patterns as well as of heterogeneity in these patterns across study populations. In line with previous research, I visualize all main results in Figures 1, 2 and 3.

In addition to these main analyses, three sets of additional models have been performed. First, the role of changes in individual BMI for ideal weight was assessed in models controlling for individual BMI (M1c and M1d, Table 2). Second, the role of BMI in reference groups for ideal weight was assessed in M1e and M1f (Table A4 in Appendix), which controlled for both individual and reference group BMI. Finally, demographic indicators for education, immigrant status and civil were included into the models M1g and M1h (Table A4 in Appendix) for ideal weight and into the models for weight discrepancy M2c, M2d, M3c and M3d, as previous research has

suggested that weigh perceptions may vary between demographic groups independently of differences between these groups in BMI (20). The results of these additional analyses are presented in Tables 2 and 3 (main text), A4 and A5 (Appendix), described in the results section and are visualized in Figures 1 and 2 (main text) and Figures A1 and A2 (Appendix).

Supplement 2: Control variables

Education was measured as highest level of education attended, distinguishing between (1) primary and intermediate secondary education, (2) higher secondary and intermediate vocational education, (3) higher vocational education, and (4) university education and higher. Migration status indicator distinguished between those with migration background – including (1) 1st generation migrants (not born in the Netherlands) and (2) 2nd generation migrants (being born in the Netherlands but having either mother or father who were not born in the Netherlands), and (3) those without migration background (born in the Netherlands and both parents born in the Netherlands). The indicator of civil status distinguished between four categories: (1) married, (2) separated or divorced, (3) widowed, (4) never been married.

Appendix 2:

Table A1. *Age and Cohort Overlaps in the Analytic Sample*

	Age													
Cohort	16-20	21-25	26-30	31-35	36-40	41-45	46-50	51-55	56-60	61-65	66-70	71-75	76-80	Total
1930-39	0	0	0	0	0	0	0	0	0	0	348	1,431	1,738	3,517
1940-49	0	0	0	0	0	0	0	0	696	3,397	4,505	2,323	387	11,308
1950-59	0	0	0	0	0	0	753	3,267	4,991	3,070	744	0	0	12,825
1960-69	0	0	0	0	781	3,213	4,572	2,624	628	0	0	0	0	11,818
1970-79	0	0	575	2,591	3,833	2,192	486	0	0	0	0	0	0	9,677
1980-89	514	1,746	2,690	1,691	430	0	0	0	0	0	0	0	0	7,071
1990-99	3,010	1,721	416	0	0	0	0	0	0	0	0	0	0	5,147
2000-02	326	0	0	0	0	0	0	0	0	0	0	0	0	326
Total	3,850	3,467	3,681	4,282	5,044	5,405	5,811	5,891	6,315	6,467	5,597	3,754	2,125	61,689

Note. Data from 12 waves of the Longitudinal Internet Studies for the Social Sciences (LISS) collected between 2007 and 2018. Number of observations is presented in the cells.

Table A2: Attrition analyses (Women)

	Left before 2018				Stayed until 2018			
	Mean	SD	Min	Max	Mean	SD	Min	Max
Age	44.71	17.15	16	80	49.69	16.14	16	80
Year of birth	1967	17.52	1927	2001	1967	18.05	1927	2002
BMI	25.03	5.061	12.11	81.37	25.45	4.93	10.98	69.92
Ideal BMI	23.14	3.33	11.71	73.58	23.58	3.31	10.40	73.46
Weight discrepancy (kg/m2)	2.10	2.78	0	51.07	2.04	2.83	0	42.24
Current weight = Ideal weight	.18		0	1	.17		0	1
Current weight > Ideal weight	.75		0	1	.77		0	1
Current weight < Ideal weight	.07		0	1	.05		0	1
Number of waves	3.63	2.91	1	11	7.65	4.26	1	12
N individuals (Observations)				4,314 (13,606)				2,955 (19,699)

Note. Data from 12 waves of the Longitudinal Internet Studies for the Social Sciences (LISS) collected between 2007 and 2018.

Table A3: Attrition analyses (Men)

	Left before 2018				Stayed until 2018			
	Mean	SD	Min	Max	Mean	SD	Min	Max
Age	45.90	17.91	16	80	52.43	15.64	16	80
Year of birth	1966	18.14	1927	2001	1964	17.84	1927	2002
BMI	25.39	4.14	14.93	64.27	25.90	3.88	13.88	64.40
Ideal BMI	24.24	2.77	14.69	71.66	24.58	2.66	13.23	88.16
Weight discrepancy (kg/m2)	1.60	2.28	0	54.88	1.57	2.08	0	65.31
Current weight = Ideal weight	.22		0	1	.20		0	1
Current weight > Ideal weight	.62		0	1	.69		0	1
Current weight < Ideal weight	.15		0	1	.10		0	1
Number of waves	3.42	2.85	1	11	8.07	4.18	1	12
N individuals (Observations)			3,646 (10,895)				2,494 (17,589)	

Note. Data from 12 waves of the Longitudinal Internet Studies for the Social Sciences (LISS) collected between 2007 and 2018.

Appendix 3: HLM results

Table A4: Hierarchical Linear Regression Models for Change in Ideal BMI for men and women

	M1a<br>Ideal BMI<br>(kg/m2)<br>Women	M1b<br>Ideal BMI<br>(kg/m2)<br>Men	M1c<br>Ideal BMI<br>(kg/m2)<br>Women	M1d<br>Ideal BMI<br>(kg/m2)<br>Men	M1e<br>Ideal BMI<br>(kg/m2)<br>Women	M1f<br>Ideal BMI<br>(kg/m2)<br>Men	M1g<br>Ideal BMI<br>(kg/m2)<br>Women	M1h<br>Ideal BMI<br>(kg/m2)<br>Men
Age	0.735***<br>[0.657,0.812]	0.447***<br>[0.374,0.519]	0.402***<br>[0.343,0.461]	0.218***<br>[0.165,0.271]	0.407***<br>[0.349,0.466]	0.220***<br>[0.167,0.273]	0.414***<br>[0.354,0.475]	0.233***<br>[0.179,0.288]
Age2	-0.849***<br>[-1.263,-0.436]	-1.180***<br>[-1.562,-0.798]	-0.017<br>[-0.338,0.305]	-0.449**<br>[-0.740,-0.157]	-0.089<br>[-0.413,0.235]	-0.578***<br>[-0.877,-0.279]	-0.018<br>[-0.356,0.320]	-0.517**<br>[-0.831,-0.203]
Cohort	0.261***<br>[0.187,0.335]	0.254***<br>[0.188,0.320]	0.042<br>[-0.012,0.097]	0.091***<br>[0.041,0.140]	0.067*<br>[0.011,0.124]	0.116***<br>[0.065,0.168]	0.126***<br>[0.067,0.186]	0.145***<br>[0.091,0.199]
Cohort * Age	-0.061*<br>[-0.116,-0.005]	-0.018<br>[-0.068,0.031]	-0.031<br>[-0.068,0.005]	-0.050**<br>[-0.083,-0.017]	-0.045*<br>[-0.082,-0.008]	-0.078***<br>[-0.114,-0.042]	-0.039<br>[-0.078,0.000]	-0.073***<br>[-0.111,-0.035]
Cohort * Age2	-0.334***<br>[-0.433,-0.234]	-0.446***<br>[-0.536,-0.357]	-0.091**<br>[-0.154,-0.028]	-0.148***<br>[-0.205,-0.092]	-0.079*<br>[-0.142,-0.016]	-0.138***<br>[-0.194,-0.081]	-0.080*<br>[-0.148,-0.012]	-0.116***<br>[-0.175,-0.056]
Individual BMI			0.456***<br>[0.450,0.461]	0.468***<br>[0.461,0.475]	0.455***<br>[0.449,0.460]	0.466***<br>[0.460,0.473]	0.450***<br>[0.444,0.456]	0.468***<br>[0.461,0.475]
Reference group BMI					0.049***<br>[0.021,0.077]	0.060***<br>[0.028,0.091]	0.056***<br>[0.027,0.085]	0.061***<br>[0.027,0.094]
Education (ref. University)								
<= Interm. secondary							0.497***<br>[0.374,0.620]	0.355***<br>[0.252,0.459]
Higher sec./ interm. voc.							0.233***<br>[0.124,0.342]	0.256***<br>[0.165,0.347]
Higher voc.							0.151**<br>[0.042,0.261]	0.062<br>[-0.029,0.154]

Continued on the next page

Table A4 continued

Immigrant status (ref. non-migrant)								
1st generation migrant							-0.114 [-0.298,0.070]	0.297*** [0.135,0.459]
2nd generation migrant							-0.040 [-0.142,0.062]	0.037 [-0.059,0.133]
Civil stat. (ref. married)								
Divorced/separated							-0.427* [-0.769,-0.085]	-0.002 [-0.339,0.336]
Widowed							-0.083 [-0.188,0.022]	0.019 [-0.081,0.118]
Never married							-0.086 [-0.235,0.064]	-0.076 [-0.250,0.097]
Intercept	23.648*** [23.557,23.739]	24.818*** [24.739,24.898]	23.408*** [23.361,23.454]	24.476*** [24.432,24.519]	22.145*** [21.426,22.865]	22.900*** [22.072,23.729]	21.749*** [20.989,22.509]	22.701*** [21.825,23.576]
N	33,085	28,346	33,085	28,346	33,085	28,346	31,696	27,111

Note. Data from 12 waves of the Longitudinal Internet Studies for the Social Sciences (LISS) collected between 2007 and 2018. Age, Age2, Cohort were centered at sex-specific means and divided by 10 for better readability of the coefficients. Individual and reference group BMI was centered at sex-specific means; 95% confidence intervals in brackets, * $p < 0.05$, ** $p < 0.01$, *** $p < 0.001$

Table A5 Hierarchical Linear Regression Models for Change in Weight Discrepancy for Men and Women with Controls

	M2a Weight discr.(kg/m2) Women	M2b Weight discr.(kg/m2) Men	M2c Weight discr.(kg/m2) Women	M2d Weight discr.(kg/m2) Men	M3a Weight discr.(%) Women	M3b Weight discr.(%) Men	M3c Weight discr.(%) Women	M3d Weight discr.(%) Men
Age	-0.014	0.114***	-0.004	0.094**	-0.248*	0.185	-0.223	0.126
	[-0.090,0.063]	[0.055,0.173]	[-0.084,0.076]	[0.033,0.155]	[-0.479,-0.017]	[-0.007,0.378]	[-0.461,0.015]	[-0.073,0.324]
Age2	-0.788***	-0.558***	-0.979***	-0.512***	-2.577***	-1.481***	-3.014***	-1.324***
	[-1.219,-0.356]	[-0.745,-0.372]	[-1.437,-0.520]	[-0.710,-0.315]	[-3.881,-1.273]	[-2.101,-0.861]	[-4.376,-1.651]	[-1.972,-0.676]
Cohort	0.174***	0.156***	0.246***	0.172***	0.385***	0.511***	0.582***	0.547***
	[0.099,0.249]	[0.092,0.220]	[0.166,0.325]	[0.105,0.239]	[0.164,0.607]	[0.307,0.715]	[0.348,0.815]	[0.333,0.761]
Cohort2		-0.107		-0.164		-0.015		-0.322
		[-0.314,0.101]		[-0.393,0.065]		[-0.690,0.659]		[-1.052,0.408]
Cohort * Age	-0.013		-0.042		-0.091		-0.162*	
	[-0.063,0.038]		[-0.097,0.013]		[-0.240,0.058]		[-0.322,-0.002]	
Cohort * Age2	-0.151**		-0.159**		-0.265		-0.314*	
	[-0.241,-0.060]		[-0.258,-0.059]		[-0.532,0.002]		[-0.602,-0.025]	
Education (ref. Univ.)								
<= Interm. secondary			0.852***	0.473***			2.182***	1.316***
			[0.639,1.066]	[0.323,0.623]			[1.586,2.779]	[0.869,1.763]
Higher sec../ interm. voc.			0.721***	0.351***			1.865***	1.007***
			[0.524,0.919]	[0.221,0.481]			[1.312,2.418]	[0.607,1.407]
Higher voc.			0.407***	0.187**			1.115***	0.556**
			[0.208,0.607]	[0.056,0.319]			[0.555,1.675]	[0.151,0.961]
Immigrant status (ref. non-migrant)								
1st generation migrant			0.414**	0.325**				
			[0.101,0.726]	[0.085,0.564]				
2nd generation migrant			0.540***	0.236**				
			[0.360,0.720]	[0.094,0.379]				

Continued on the next page

***Table A5 continued*.**

Civil status (ref. Married)								
Divorced/separated			-0.064	-0.498*			-0.523	-1.236
			[-0.520,0.393]	[-0.936,-0.059]			[-1.874,0.828]	[-2.635,0.163]
Widowed			0.044	-0.080			0.093	-0.065
			[-0.114,0.202]	[-0.221,0.060]			[-0.357,0.542]	[-0.491,0.362]
Never married			0.045	0.093			0.120	0.432
			[-0.157,0.247]	[-0.147,0.332]			[-0.459,0.700]	[-0.270,1.133]
Intercept	2.289***	1.823***	1.620***	1.546***	7.738***	6.217***	5.974***	5.405***
	[2.211,2.367]	[1.758,1.888]	[1.433,1.808]	[1.426,1.666]	[7.524,7.953]	[6.022,6.412]	[5.449,6.498]	[5.038,5.771]
N	33,085	28,346	33,085	28,346	33,085	28,346	31,696	27,111

Note. Data from 12 waves of the Longitudinal Internet Studies for the Social Sciences (LISS) collected between 2007 and 2018. Age, Age2, Cohort were centered at sex-specific means and divided by 10 for better readability of the coefficients. 95% confidence intervals in brackets, * $p < 0.05$, ** $p < 0.01$, *** $p < 0.001$

Table A6: HLPM Models for Change in Categories of Weight Discrepancy for Men and Women

	M4a<br>Ideal BMI = Current BMI	M4b<br>Ideal BMI = Current BMI	M5a<br>Ideal BMI < Current BMI	M5b<br>Ideal BMI < Current BMI	M6a<br>Ideal BMI > Current BMI	M6b<br>Ideal BMI > Current BMI
Age	0.005	-0.011	-0.008	0.030***	0.002	-0.015**
	[-0.006,0.016]	[-0.024,0.001]	[-0.019,0.003]	[0.017,0.043]	[-0.004,0.009]	[-0.026,-0.004]
Age2	0.062	0.115***	-0.131***	-0.255***	0.066***	0.113***
	[-0.005,0.129]	[0.091,0.139]	[-0.168,-0.094]	[-0.296,-0.214]	[0.043,0.089]	[0.052,0.173]
Cohort	-0.005	-0.013	0.003	0.004	0.002	0.001
	[-0.016,0.007]	[-0.025,0.000]	[-0.009,0.014]	[-0.010,0.017]	[-0.005,0.009]	[-0.009,0.010]
Cohort2			-0.016	-0.069**	0.006	
			[-0.056,0.025]	[-0.116,-0.023]	[-0.017,0.030]	
Cohort * Age	-0.001					-0.008*
	[-0.008,0.006]					[-0.015,-0.002]
Cohort * Age2						0.022***
						[0.010,0.034]
Intercept	0.158***	0.176***	0.800***	0.754***	0.042***	0.071***
	[0.149,0.168]	[0.166,0.187]	[0.789,0.811]	[0.741,0.768]	[0.037,0.047]	[0.063,0.079]
N	33085	28,346	33085	283,46	33085	28,346

Note. Data from 12 waves of the Longitudinal Internet Studies for the Social Sciences (LISS) collected between 2007 and 2018. HLPM refers to Hierarchical Linear Probability Models; Age, Age2, Cohort were centered at sex-specific means and divided by 10 for better readability of the coefficients. BMI was centered at sex-specific means¸ 95% confidence intervals in brackets, * $p < 0.05$, ** $p < 0.01$, *** $p < 0.001$

Appendix 4: Figures

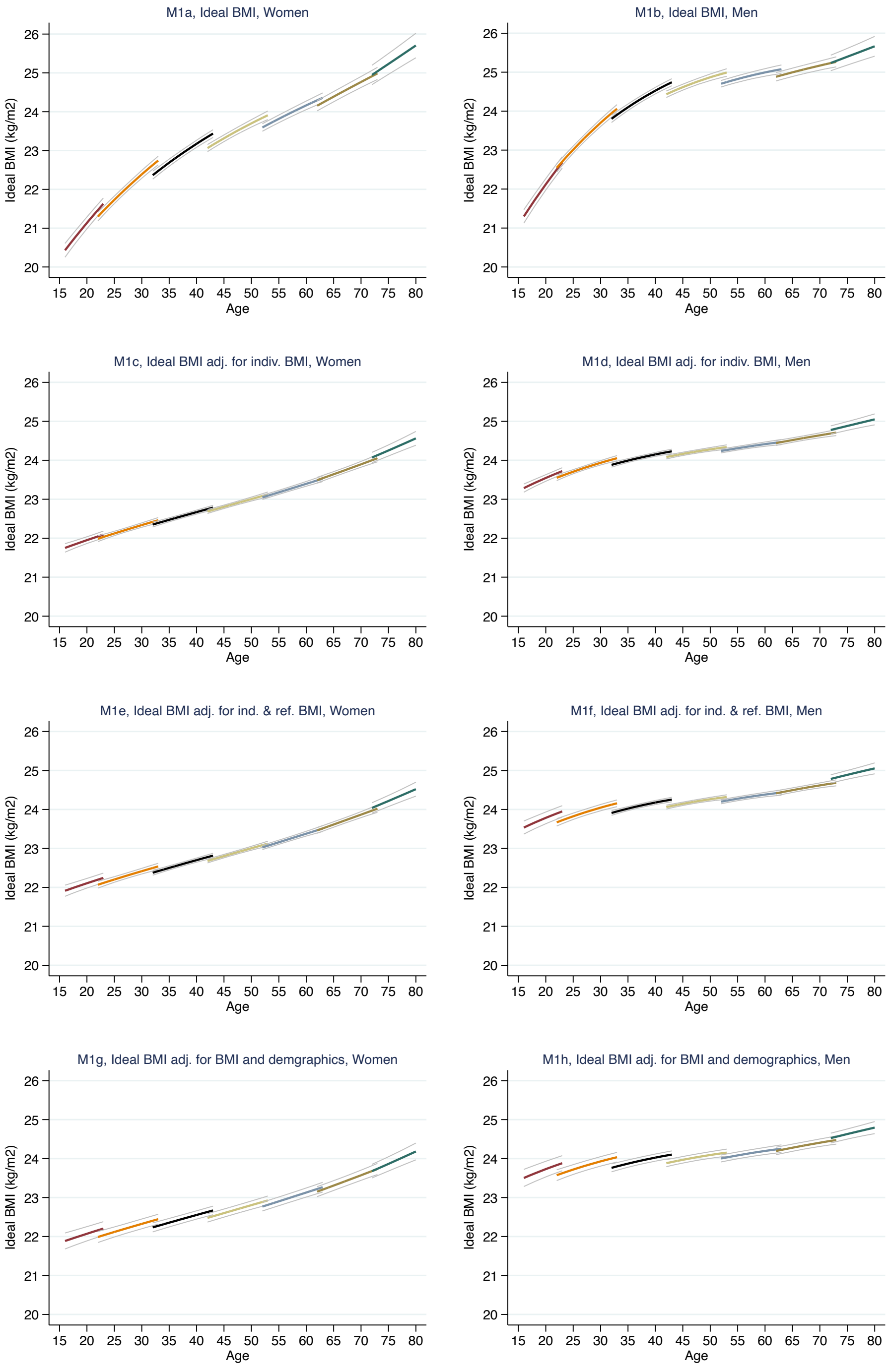

<u>FIGURE A1. LIFE COURSE AND COHORT PROFILES OF IDEAL WEIGHT</u>

Note. Data from 12 waves of the Longitudinal Internet Studies for the Social Sciences (LISS) collected between 2007 and 2018. Curves are based on models (M1a-M1d) shown in Table 2 and Table A4. In the plots visualizing M1c-M1f indiv. BMI and reference group BMI were fixed at the sex specific means. In the plots visualizing M1g and M1h, education was fixed at "University"; immigrant status was fixed at "non-migrant"; civil status was fixed at "Married".

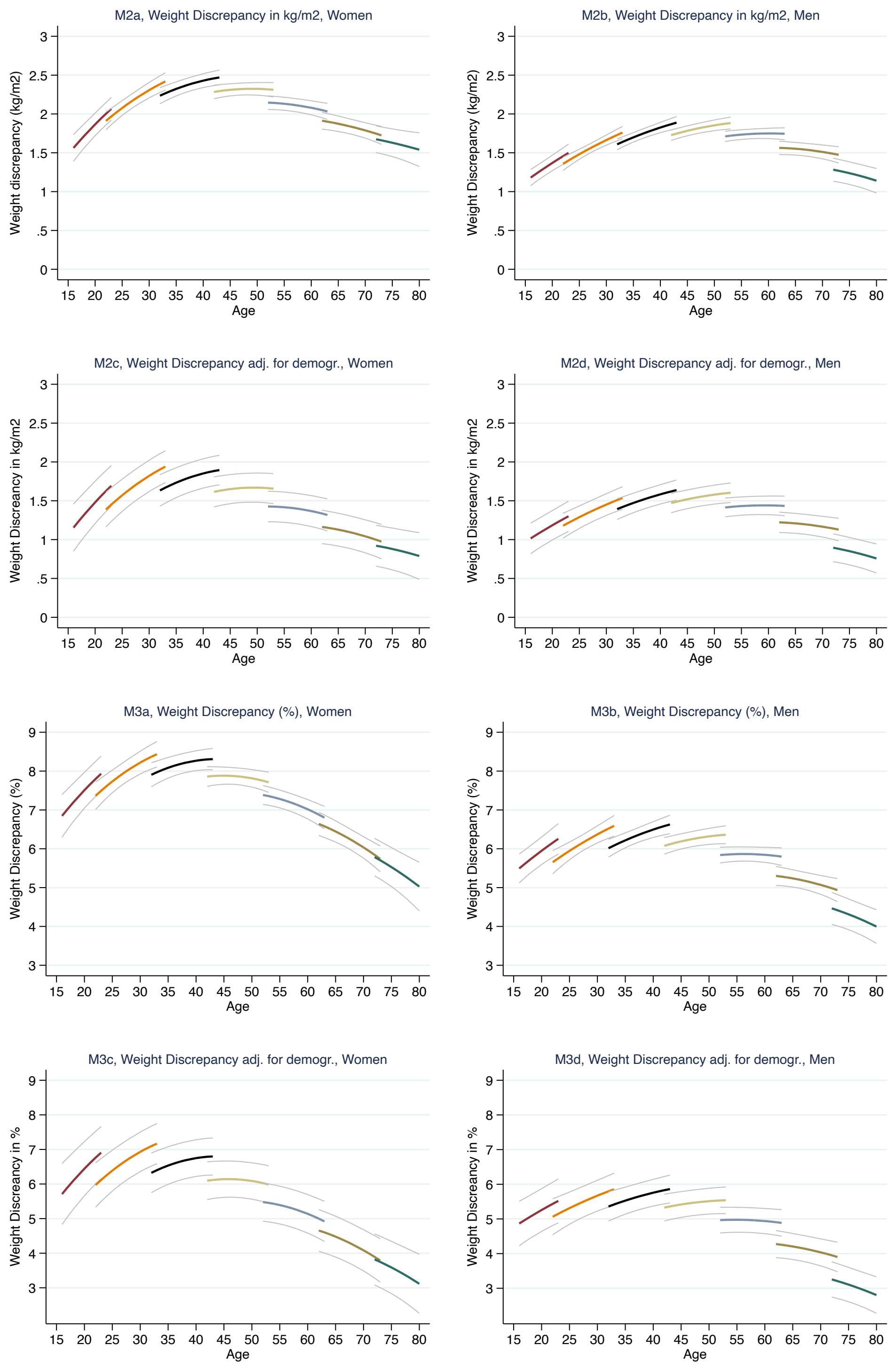

FIGURE A2. LIFE COURSE AND COHORT PROFILES OF WEIGHT DISCREPANCY

Note. Data from 12 waves of the Longitudinal Internet Studies for the Social Sciences (LISS) collected between 2007 and 2018. Curves are based on models shown in Table 3 M2a, M2b, M3a, M3b) and Table A5 (M2c, M2d, M3c, M3d). In the plots visualizing M2c and M2d as well as M3c and M3d, education was fixed at "University"; immigrant status was fixed at "non-migrant"; civil status was fixed at "Married".
